# Policy dialogues – the “bolts and joints” of policy-making: experiences from Cabo Verde, Chad and Mali

**DOI:** 10.1186/s12913-016-1455-x

**Published:** 2016-07-18

**Authors:** Delanyo Dovlo, Juliet Nabyonga-Orem, Yolanda Estrelli, Aziza Mwisongo

**Affiliations:** 1 Health Systems and Services Cluster, World Health Organization Regional Office for Africa, Cite de Djoue, BP 06, Brazzaville, Republic of Congo; 2 Health Systems and Services, World Health Organization Cabo Verde Country Office, PO Box 266, Praia, Cabo Verde

**Keywords:** Health policy dialogue, Policy-making, Policy processes, Policies, Strategies

## Abstract

**Background:**

Policy processes that yield good outcomes are inherently complex, requiring interactions of stakeholders in problem identification, generation of political will and selection of practical solutions. To make policy processes rational, policy dialogues are increasingly being used as a policy-making tool. Despite their increasing use for policy-making in Africa, evidence is limited on how they have evolved and are being used on the continent or in low and middle income countries elsewhere.

**Methods:**

This was an exploratory study using qualitative methods. It utilised data related to policy dialogues for three specific policies and strategies to understand the interplay between policy dialogue and policy-making in Cabo Verde, Chad and Mali. The specific methods used to gather data were key informant interviews and document review. Data were analysed inductively and deductively using thematic content analysis.

**Results:**

Participation in the policy dialogues was inclusive, and in some instances bottom-up participatory approaches were used. The respondents felt that the execution of the policy dialogues had been seamless, and the few divergent views expressed often were resolved in a unanimous manner. The policies and strategies developed were seen by all stakeholders as relating to priority issues. Other specific process factors that contributed to the success of the dialogues included the use of innovative approaches, good facilitation, availability of resources for the dialogues, good communication, and consideration of the different opinions. Among the barriers were contextual issues, delays in decision-making and conflicting coordination roles and mandates.

**Conclusions:**

Policy dialogues have proved to be an effective tool in health sector management and could be a crucial component of the governance dynamics of the sector. The policy dialogue process needs to be institutionalised for continuity and maintenance of institutional intelligence. Other essential influencing factors include building capacity for coordination and facilitation of policy dialogues, provision of sustainable financing for execution of the dialogues, use of inclusive and bottom-up approaches, and timely provision of reliable evidence. Ensuring continued participation of all the actors necessitates innovation to allow dialogue outside the formal frameworks and spaces that should feed into the formal dialogue processes.

## Background

Policy processes that yield good outcomes are inherently complex, requiring interactions of stakeholders in problem identification, generation of political will and selection of practical solutions [1]. Policy-making processes in low and middle income countries are criticised for being prescriptive, insufficiently evidence-based and inconsiderate of the underlying contexts [2]. Globally, and particularly in Africa, policy dialogues have been recently recognised as an essential part of policy-making processes. Rajan et al. [3] define policy dialogue as “… part and parcel of policy and decision-making processes, where they are intended to contribute to developing or implementing policy change following a round of evidence-based discussions/workshops/consultations on a particular subject”.

The literature identifies a number of barriers to the use of evidence during policy-making [2, 4], among which are how the research is conducted and how the information is gathered, packaged and presented, as well as the complexity of the environment in which the evidence is used and applied during policy-making processes [2, 4]. But even with the availability of evidence, challenges still remain in making effective policies and decisions [4]. Innovative knowledge translation measures such as those that bridge the gap between policy-makers and researchers, and researchers’ efforts to transform research findings into easier and non-scientific language have been helpful [4, 5]. However, some authors argue that these efforts only solve part of the problem and that policy-making processes are complex and are often driven by political factors rather than scientific evidence [2, 4].

The linear model of policy-making, which theorises that policy processes are rational, balanced, objective and analytical [6, 7], has been used extensively to understand the policy process and its key phases [6]. Several criticisms have been advanced against the concept of that model, with some critics arguing that the policy process is highly political and messy and often has strong influence of a few powerful actors and elites [8, 9]. Further, forums do not exist where stakeholders with different experiences can constructively debate, articulate their ideas and share scientific evidence to generate realistic policy decisions and foster their implementation [10]. According to Thomas and Grindle [7], the policy-making process involves agenda-setting, decision and implementation phases. Several authors [11, 12] argue for the involvement of the key stakeholders such as frontline implementers in the initial phases of policy-making such as the agenda-setting and decision phases, since the ultimate responsibility of implementation of the policies will fall on them. These implementers have the power to alter or implement a policy based on contextual factors [11, 12].

Despite its criticism, the linear model identifies the key elements within the phases of the policy process, which are (1) recognising and defining the nature of the issue to be dealt with, (2) identifying the possible courses of action to deal with the issue, (3) weighing up the advantages and disadvantages of each alternative, (4) choosing the option that offers the best solution, (5) implementing the policy, and (6) evaluating the outcome [7]. Dialogue among stakeholders needs to take place in these phases of the policy process. Policy dialogues are believed to offer an opportunity for interactive knowledge sharing in policy-making arenas [3, 5, 13, 14]. They allow for several stakeholders of different calibre to come together to discuss an issue of common interest [3]. This creates buy-in and ownership of the outcome and builds commitment to its success, which consequently influences the implementation of the policy [1, 3, 5]. Policy dialogues galvanise the decision-making process by making it inclusive and moving it beyond the policy-making domains [3].

A policy dialogue acquires value only by the way it is conducted [3, 15]. Literature recognises the importance of how a policy dialogue is organised, in generating good decisions [3, 15, 16]. Lavis et al. [5] emphasise that a policy dialogue must have three major elements for it to be beneficial: it should provide the opportunity to discuss the problem of interest, it should consider different options for decisions, and it should chart a way forward in terms of how the outcomes will be implemented [5]. Additionally, for a policy dialogue to be considered effective, its subject or matter of interest should be shared and timely, accord with the culture or norms of the community, and be seen as a priority by all stakeholders [5].

Despite the increasing use of policy dialogues for policy-making, there is limited evidence on how they have evolved and are being used for decision-making in Africa and low and middle income countries elsewhere. One objective of this study was to review the policy dialogue processes involved in developing major policies in three African countries to relate the process of policy dialogue to the context of policy-making. Further, the study sought to explore and understand the processes involved in policy dialogue using three policy-making case studies from Cabo Verde, Chad and Mali. The policies were the National Pharmaceutical Policy in Cabo Verde; the Plan National de Développement Sanitaire (PNDS) (The National health development plans – NHDP 2) for 2012–2016 in Chad, and the Programme de Développement Sanitaire et Social (PRODESS) 2014–2018 (Health and Social Program) in Mali.

## Methods

### Study design

This was an exploratory study using qualitative methods. The design and execution of the study were guided by the main consideration in a policy dialogue developed by Rajan et al. [3]. These were detailed as: identifying entry points; how to manage a policy dialogue process and the roles of stakeholders. This study utilised data related to policy dialogues of three specific polices and strategies in understanding the interplay between policy dialogue and policy-making in Cabo Verde, Chad and Mali. These countries were chosen owing to their experiences in formulating policies using policy dialogue platforms.

### Study population

The study population included health sector actors who had been engaged in policy dialogue processes at national and sub-national levels. The initial list of respondents was drawn up in consultation with a team from the World Health Organization (WHO) office in each of the countries, who identified the stakeholders involved in the policies and strategies of interest. Additional respondents were identified using the snowballing technique until descriptive saturation was achieved [17]. Other considerations in the selection of respondents were their seniority in employment, active participation in the policy process and knowledge on the research question [17]. Table 1 shows the organisational affiliation of the respondents from the three countries.

**Table 1:** Affiliation of key respondents in Cabo Verde, Chad and Mali

Institution	Cabo Verde	Chad	Mali
National level			
Ministry of health	5	6	7
Donor agencies	1	4	2
Civil society organisations	6	0	2
Sub-national level			
Ministry of health	0	4	2
Donor agencies	0	0	
Civil society organisations	2	0	
Total	14	14	13

### Data collection methods

Data were collected through key informant interviews. An interview guide was developed with standardised questions and probes on policy dialogues related to the policy of interest in the three countries. The four areas of interest were the reasons that made the policy a priority for policy dialogue, the actors that were involved, the divergent and consensus points during the dialogue, and facilitating and barrier factors in the policy dialogue processes. In each country data were collected by an independent researcher, who was an expert in qualitative research and knowledgeable on health policy and systems. The interviews, which lasted 50 min on average, were conducted in French in Chad and Mali and Portuguese in Cabo Verde. The data were collected between June and August 2015. Both published and grey literature relating to policy dialogues and the policies of interest was reviewed for additional information.

All key informant interviews were audio-recorded and later transcribed verbatim before translation into English by the consultants. After transcription into Word, all the transcripts were exported into MAXQDA software for analysis. The authors of this paper read all the transcripts in detail to identify emerging issues in line with the study objective. Codes and sub-codes were developed both inductively and deductively from the questions. We were interested in the obvious and underlying meanings from the transcripts, which we then categorised into themes. The themes were compared among the key informants in each country and then across the countries.

## Results

The European Union, WHO and the Government of Luxembourg signed a partnership agreement in 2013 for capacity building in health policy development to achieve equitable universal health coverage in 13 countries in Africa. That agreement, called the Partnership for Universal Health Coverage, was implemented in the form of technical support for policy dialogue on health issues. The policy dialogue initiative was approached differently among the three countries, with each addressing issues in the dialogues according to its needs to support and meet the goals of universal health coverage. Some of the dialogues focused on strengthening of health planning processes, monitoring and evaluation, health financing, and improvement in alignment and harmonisation of health sector actors.

### Country contexts related to the dialogues

In 2013 the, European Union, the Government of Luxembourg and WHO entered into a collaborative agreement to support policy dialogue on national health policies, strategies and plans and universal health coverage in Cabo Verde. Since signing the agreement in May 2013, the country has undertaken several activities related to policy dialogue for health, from which two were identified as priority areas: the signing of the National Health Compact and the revision of the National Pharmaceutical Policy. The Pharmaceutical Forum was the platform used for policy dialogue in developing the pharmaceutical policy.

**Fig. 1:**
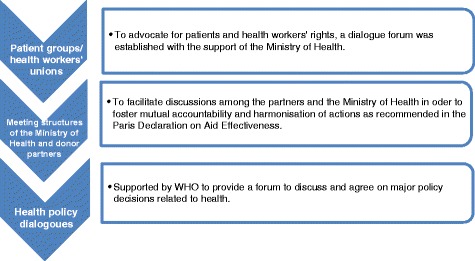
Development of the health policy dialogue in Chad

Health policy dialogue in Chad is a new trend that was born partly as a result of the strategies developed by patient organisations and professional unions with the onset of diseases such as AIDS, to advocate for patients and health workers’ rights. A dialogue forum was established by patients’ groups and health workers’ unions with the support of the Ministry of Health. To fulfil the requirements of the Paris Declaration on Aid Effectiveness a formal and dynamic consultation framework, referred to as the health policy dialogue, was established by the Ministry of Public Health. Recently, these country initiatives on policy dialogues have been supported by the European Union-Luxembourg-WHO Partnership programme. In 2013 Chad used the policy dialogue approach to develop the PNDS for 2012–2016, also called the National health development plan (NHDP). Figure 1 depicts the evolution of the policy dialogue process in Chad.

In Mali, the Partnership for Universal Health Coverage focused on intensifying support for the implementation of PRODESS for 2014–2018 through facilitation of policy dialogue. The dialogue for PRODESS was conducted by a committee of 10 national experts from the 3 ministries of health, social development, and advancement of women, children and the family, along with 5 international consultants distributed among the various areas of industry, and under the supervision of the director of Système Pastoral associé aux Cultures (SPC). That approach helped build a common understanding of the major issues in health and social development and move towards consensus among all the stakeholders, including health services users; health workers, experts, institutions and departments; political and social partners etc., on the strategic directions and the way for their implementation. Through this process people were sensitised and involved and their perceptions were recognised and taken into account in the policy decisions.

### Reasons for the development of specific policies

The three policies were identified as priorities by all stakeholders in the three countries. In Chad, the development of NHDP 1 was a controversial process characterised by misunderstanding among health professionals, administrators, support personnel, nongovernmental organisations (NGOs) and civil society organisations (CSOs). This was mainly fuelled by competition among the actors, each led by self-interest and the potential benefits they expected, as one respondent noted,

*“The process of developing the NHDP 1 was so chaotic with many misunderstandings due to personal influences.”* (National level representative, Chad)

The results of the final evaluation of NHDP 1 in November 2012 revealed the difficulties encountered in implementing and monitoring health programmes. It was imperative to start thinking about how such problems were to be addressed in NHDP 2. The NHDP 1 evaluation also recommended strengthening the involvement of the various health sector actors in the development of NHDP 2 and accommodating the needs of the regions by first drawing up the regional health development plans. It was also recommended that consensus on issues be build and alliances be forged with the private sector and CSOs. This happened in a context marked by the commitment of the higher state authorities to reduce mortality and maternal and infant morbidity.

In Cabo Verde, the demand for a revised pharmaceutical policy arose from the need to achieve the goal of universal health coverage. It was realised in the process of developing the national strategy for financing universal health coverage that access to drugs and pharmaceuticals needed to be improved. However, the pharmaceutical policy was outdated as regards to the transformation of the health sector, the country’s epidemiological profile and the organisation of the pharmaceutical sector.

### Reasons that prompted the revision of the pharmaceutical policy in Cape Verde

#### Health sector transformation

Since independence in 1975, Cape Verde has invested in health, adopting policies that allow and guarantee access and equity in the provision of health care. The National Pharmaceutical Policy as an integral part of the National Health Policy dates back to 2003. Since then, the sector has gone through many transformations, hence the necessity to update the document in line with the current national and international needs. According to the General Direction of Pharmacies at the Ministry of Health it was necessary to update the policy as many changes had occurred since the current policy was introduced in 2003.

#### Epidemiological change

The epidemiological profile of the country has changed since 2008. Cape Verde has become upgraded to a middle income country, even though infectious conditions such as diarrhoea are still prevalent and non-communicable diseases are increasing. The types of medicines, treatment approaches and technologies *also* have changed. Moreover, the sector is constantly developing. There are more pharmacies, professionals and health centres. The conditions and infrastructure also have changed.

#### Organisational change

In 2005, the Food and Product Regulation and Supervision Agency was established and the technical roles related to the authorisation and evaluation of medicines were transferred to it by the Ministry of Health. However, according to ARFA, the roles were not well defined, as the Ministry of Health still had a commission that verified the work of ARFA. This duplication needed to be addressed and the roles clearly defined and updated in the national pharmaceutical policy.

These reasons made the revision of the policy necessary. The development of a new national pharmaceutical policy was a priority and had the necessary political backing.

In Mali, the lack of alignment and harmonisation of health partners was a major stumbling block for health development. In the midst of this poor coordination, Mali was experiencing high infant morbidity and maternal morbidity and mortality due to communicable and non-communicable diseases, poor quality of health services, disparities in health and inequitable distribution of health services. Some part of the country was also in the midst of armed emergency and therefore, policy dialogues were an important step towards harmonisation and coordination of health stakeholders in a difficult environment.

### Processes and actors in the policy dialogues

A systematic and participatory process was employed in the policy dialogues in the three countries. In Chad the dialogues involved several steps that can be summarised as initial data gathering, advocacy and meetings. The key activities in the policy dialogue for the development of NHDP 2 were.

1. Setting up of a technical committee composed of Ministere de la Sante Publique (MSP) executives and partners with the role of mobilising resources, and development of draft documents and organisation of their validation and adoption. In addition, six thematic committees were established to analyse the situation and programming assistance for each of the six pillars of Plan National de Développement Sanitaire (PNDS).
2. Sharing information/evidence: The preliminary study reports were shared among the participants.
3. Development of regional plans: A key step was the development of 23 Plan Regionaux de development Sanaitaires (PRDS) by Developpement Sanitaire Regionaux (DSR) teams supported by central level executives. This work was also done through several stages: (1) development, validation and provision of canvas DSR and an array of delegates indicative funds (2013–2015) for each region; (2) development of the first versions of the PRDS; (3) peer review and executives from the central level; (d) validation teams DSR supported by the central level; (e) adoption of the PRDS in areas under the chairmanship of governors.
4. Development and validation of a road map.

The respondents in Cabo Verde believed that the involvement of all the relevant actors in the policy dialogue for strengthening the national pharmaceutical policies was perceived as successful. The participation of the Ministry of Health in organising the National Pharmaceutical Forum that congregated health professionals, pharmaceutical professionals, collaborators, medication authorities, NGOs, CSOs and national departments, was considered by the respondents as the main factor in the forum’s success, as one respondent remarked,

*“The pharmaceutical forum was a good platform to conduct the policy dialogues for the pharmaceutical policy.”* (National level representative, Cabo Verde)

The first National Pharmaceutical Forum organised by the Direção General de Farmacias e Medicamentos of the Ministry of Health in Cabo Verde, was held in November 2014. It involved the whole health sector and aimed to share knowledge and exchange experiences. It also served as a launching pad for the much-needed revision of the National Pharmaceutical Policy.

In Mali the process of developing the policy involved close collaboration with development partners, CSOs, government departments, key public and private players, the three relevant ministries (health and sanitation; the ministry of solidarity, humanitarian action and reconstruction of the North and; the ministry of promotion of women, children and family). As one of the respondent noted below.

*“A number of stakeholders were involved in the development of the plan, the process was very exhaustive and involved a number of dialogues”.* (NGO representative, Mali)

The process for developing the components of PRODESS in Mali adopted bottom-up and participatory approaches and was coordinated by the Director of the Unit of Planning and Statistics, Health Sector, Social Development and Promotion of the Family (Directeur de l'Unité de planification et des statistiques, secteur de la santé, du développement social et de promotion de la famille). This unit facilitated consultations in the districts and regions involving the participation of all stakeholders in the health sector, taking into account not only the problems of the community but also the determinants of health.

### Dispute areas during the dialogue process

The respondents expressed the view that the three policy dialogue processes provided room for participation and consensus building but there were challenges as well. In Chad the policy dialogue process was regarded by the respondents as having been smooth with limited disputes. The contestations were mainly around the differences between government and United Nations Children’s Fund data sets used in making decisions, as noted by one respondent,

*“There is always a problem with data, as UN agencies will come with their data, which are different from government data and that sparks debates and delays. However, in the end it was unanimously agreed to use the Ministry of Health data.”* (Donor representative, Chad)

The respondents in Cabo Verde attributed the disputes to the way the forums were structured to provide information through presentations rather than to create an environment stimulating discussion and debate. This limited debates, divergences and compromises, as was noted by the respondents. A few issues were a source of contests among the dialogue participants in Cabo Verde, one of which related to the duplication of structures and organisations with similar roles in the regulation of the pharmaceutical industry. Even though the Agência de Regulação e Supervisão de Produtos Farmacêuticos e Alimentares (ARFA) was created for this specific purpose, the Direcção General de Farmácia (DGF) still had a commission to verify ARFA work. According to the respondents, much of the disagreement during the policy dialogue related to clarification of roles of pharmaceutical actors. Another area of disagreement was the restriction by the government of the sale of non-generic drugs. This was not popular with the private pharmaceutical suppliers, who wanted to sell other more expensive and lucrative brands. As part of the policy dialogue, the government agreed to offer incentives for pharmacies to sell generic products along with other brands. On this issue, one respondent remarked,

*“The Ministry of Health had to agree to the proposal of private pharmacists to sell brand drugs, after all, private pharmacists were an important stakeholder in the development of the policy.”* (National level interviewee, Cabo Verde)

In Mali, the groups involved in the development of PRODESS 3 had divergent views on some issues. In determining priorities for the dialogue process each group wanted their area of focus to be accorded importance. Analysis of the problems and determination of how these affected the communities facilitated consensus building.

### Factors facilitating and hindering policy dialogues

#### Enabling factors

The respondents cited several factors that they considered to have facilitated the dialogue processes. In Chad trust and legitimacy of the process were regarded as key success factors in the development of NHDP 2, as one of the sub-national respondents stated,

*“The process took into account of regional differences and came up with strategic solutions for each region. This helped to perceive the plan as a realistic document rather than just another document.”* (Sub-national respondent)

Another success factor in the policy dialogue in Chad was the choice of the facilitator, who had the necessary expertise to lead the interactions among the stakeholders. WHO played an enabling role by providing national and international experts familiar with the issues of focus to support the policy dialogue processes.

In Cabo Verde the meetings accommodated the different perspectives and views from the stakeholders, allowing sensitisation and involvement of all the actors, and recognition and consideration of their interests in the decisions.

In Mali also, the dialogues were considered to have been inclusive, as noted by a respondent,

*“The possibility for each participant to contribute to the dialogue and especially realism and respect for commitments are factors that favoured consensus.”* (National level respondent, Mali)

The availability and use of evidence collected through transparent processes were appreciated by the respondents in the three countries. Further, the dialogues themselves involved the development of new supportive mechanisms and structures to facilitate information gathering. In Mali, several mechanisms were set up such as the PRODESS monitoring committee chaired by the ministers of health; social development; and promotion of women, children and family, with representation from the vice president’s office and the Technical and Financial Partners (TFP) civil society. The committee was responsible for reviewing and validating the regional plans for health and social development and monitoring their implementation. One of the respondents noted that,

*“The process of gathering information from the sub-national levels to the central level for the national plan was transparent and this built confidence among us. It also contributed to the availability of information for the dialogues.”* (National level respondent, Mali)

In the three countries the dialogues involved stakeholders from different sectors and levels. This contributed to the development of plans and strategies that went beyond the boundaries of the health sector to address the social determinants of health and the interaction between the health sector and other relevant sectors. In Cabo Verde innovative approaches were used to better advocate for and communicate about the dialogues among the different stakeholders. Some of these approaches were websites and newsletters. These helped to mobilise stakeholders.

In Chad the leadership of WHO helped coordinate and galvanise donors under one framework through their signing of a pre-agreement. The pre-agreement emphasised on alignment and harmonisation which helped to normalise the different perspectives brought to the dialogue. The policy dialogues were also used to address some previous misunderstandings and confusion. For example, in Chad a misunderstanding between the Ministry of Finance and the Ministry of Health with respect to external funding was thoroughly discussed and resolved as part of the policy dialogues.

#### Barriers

Several barriers hampered the conduct of the policy dialogues in the three countries. In Chad the Ministry of Health’s slow decision-making affected a number of elements related to the dialogue process. This was compounded by the weak skills of the managers in quality monitoring, supervision and control. During the earlier policy dialogue days in Chad, the failure to coordinate the dialogues, notably due to the unavailability of appropriate frameworks, resulted in delays.

In Mali the political turmoil and health crises, particularly the Ebola epidemic, were barriers to the process of the health policy dialogues. In all the three countries there were instances where the actors differed in their perception of the priority areas for focus mainly influenced by their parochial interests and perceived benefits from the process. This caused delays in the policy dialogue process.

Some respondents believed that clarity over the jurisdiction of the policy dialogue process was an important factor in its success. In Chad, for example, there was confusion about which institution, the Studies and Cooperation Bureau (Le Buraux des Etudes et de la Coperation) or the Ministry of Health, had responsibility over the policy dialogues. This was a constraint in conducting the dialogues.

Policy dialogues are naturally exhaustive, requiring time and investment, which can be tasking for those required to participate in them. The respondents in all the three countries lamented about the length and large number of meetings required for the policy dialogues, which resulted in poor attendance with time. In addition, limitations of the resources mainly from the central government, was a handicap in both Mali and Chad. In Chad, the perception of the policy dialogues as a programme rather than a process resulted in dependence on donors to run them.

## Discussion

From this comparative study conducted in Chad, Cabo Verde and Mali, it is evident that policy dialogues were used to develop the three policies of interest. It can be argued that the presence of the health policy dialogue programme was a catalyst in the use of policy dialogue approaches for the aforementioned policy processes. For a policy dialogue to be effectual, the topic of interest should be perceived as a high priority by concerned stakeholders so that it is accorded lengthy discussion and negotiation [3]. The three policies that are the focus of this paper were deemed necessary in their countries and this was crucial in attracting the interest of all stakeholders. In Mali the PRODESS 2014–2018 was thought to be necessary following a situational analysis of the health sector. In Chad, the need for NHDP 2 was impelled by the failure of previous policy-making and implementation processes. In Cabo Verde, the efforts to achieve the universal health coverage aims brought out the realisation that a policy was needed to guide the equitable access to drugs and pharmaceuticals.

Literature on policy dialogues and effective policy-making insists on participatory and inclusive approaches that yield consensus and create approachable policies, since such approaches take into consideration the perspectives of all the actors [1, 3]. In the three countries there was extensive and high level involvement of stakeholders during the policy dialogues. The stakeholders ranged from key policy-makers to routine policy implementers. This is laudable, as a good policy dialogue requires participation and inclusion of key stakeholders to enrich the discourse [10, 14]. The involvement of a broad range of stakeholders such as implementers and beneficiaries provides the opportunity to consider the policy of interest from various perspectives such as culturally, economically and politically, as well as to accommodate donor-vested interests, existing initiatives and opportunities. Through facilitating the accumulation of an array of experiences that help to articulate the problem and suggest solutions from several perspectives, this approach also promotes ownership, accountability and commitment to the outcome [12, 18].

Some authors criticise the differentiation of policy-making and policy implementation that regards the former as political and the latter as administrative [6, 7, 18], because these processes are not separate. In reality policy implementation begins during the agenda-setting and decision phases and as such requires the involvement of implementers then.

There is an argument that policy implementation is not rational and that alterations to a policy in the process of implementation may result in the failure to execute it as intended in the objectives [12]. Some theories such as the interactive model and street level bureaucracies [12] elucidate this complexity of implementation and the role of actors. These theories emphasise the need for involvement of implementers during policy-making processes in order to foster ownership and buy-in, forge alliances and ensure commitment to the process [5, 10].

New policies often lead to transformation, which often implies change, which in turn creates fear of the unknown or loss of position and benefits [6]. Borrowing from management theories, some authors propose that fear of change needs to be managed early enough during policy-making processes [6]. This will help to avoid unintended negative consequences, as they are mitigated during participatory policy dialogues [6]. Our study shows how the fear by the private pharmaceutical companies in Cabo Verde of the consequences that could arise from the development of a pharmaceutical policy was mitigated by their involvement in the policy dialogue. The policy dialogue process, through its participatory nature, is one way of managing change. The dialogue process allowed the participants to raise their concerns, which were considered and accommodated in the policy decisions, allowing the new policy to be seen as an opportunity rather than a threat [6]. In another situation also in Cabo Verde, the pharmacists’ resistance to selling generic drugs was addressed during the development of the pharmaceutical policy. In both Mali and Chad interdepartmental differences were resolved in smaller group dialogues specially set up as part and parcel of the overall policy dialogue processes. It can be assumed that tackling these tensions during the policy-making stage would reduce the possibility of resistance to the policies during their implementation. It is also clear that these tensions were prompted by underlying professional or departmental interests among the actors and an important factor towards good policy-making and implementation [8, 9, 11, 12].

We see from our study that some specific process factors helped make the dialogues successful. These included the use of innovative and bottom-up approaches, availability of resources, good communication, and consideration of the different opinions. These are what policy dialogues experts insist are the key factors for successful dialogue processes [3, 14, 19]. Several health systems’ factors also played a major role in enabling the policy dialogues. These were harmonisation of partners, multisectoral approaches, supportive structures, and partnerships. The literature on policy-making makes a strong association between good health systems’ indicators and good policies.

## Conclusion

In Cabo Verde, Chad and Mali policy dialogues were instrumental in developing three important policies and in strengthening policy-making processes. They proved to be an effective management tool in the health sector and could be integrated as a component of the dynamics of governance of the sector. It is recommended that similar dialogues be instituted in the development of other polices, plans and strategies. To maximise the benefits from policy dialogues the following actions are necessary:

- Develop capacity for coordinating, conducting and facilitating policy dialogues in each of the countries, and clearly articulate the coordination roles;
- Provide resources for the execution of proper policy dialogues, ensuring they utilise inclusive bottom-up approaches;
- Enhance the reliability of and trust in data, which will strengthen the legitimacy of and trust in the dialogues;
- Innovatively design and allow other dialogue mechanisms outside the formal frameworks but these should feed into the formal dialogue processes;
- Institutionalise policy dialogues as part of routine governance processes that help to maintain institutional continuity and intelligence.

## Abbreviations

ARFA, Agência de Regulação e Supervisão de Produtos Farmacêuticos e Alimentares; CSOs, civil society organisations; NGOs, nongovernmental organisations; NHDP, National Health Development Plan; PRODESS, Plan décennal de développement sanitaire et social; Programme de Développement Sanitaire et Social; WHO, World Health Organization.
